# Unraveling the Bone Tissue Microenvironment in Chronic Lymphocytic Leukemia

**DOI:** 10.3390/cancers15205058

**Published:** 2023-10-19

**Authors:** Paolo Giannoni, Cecilia Marini, Giovanna Cutrona, Gian Mario Sambuceti, Franco Fais, Daniela de Totero

**Affiliations:** 1 Department of Experimental Medicine, Biology Section, University of Genova, 16132 Genova, Italy; paolo.giannoni@unige.it; 2 Nuclear Medicine Unit, IRCCS Ospedale Policlinico San Martino, 16132 Genova, Italy; cecilia.marini@unige.it (C.M.); gianmario.sambuceti@unige.it (G.M.S.); 3 CNR Institute of Bioimages and Molecular Physiology, 20054 Milano, Italy; 4 Molecular Pathology Unit, IRCCS Ospedale Policlinico San Martino, 16132 Genova, Italy; giovanna.cutrona@hsanmartino.it (G.C.); franco.fais@unige.it (F.F.); 5 Department of Health Sciences, University of Genova, 16132 Genova, Italy; 6 Department of Experimental Medicine, Anatomy Section, University of Genova, 16132 Genova, Italy

**Keywords:** bone tissue, chronic lymphocytic leukemia, tumor microenvironment

## Abstract

**Simple Summary:**

Bone remodeling requires a delicate balance between bone-forming osteoblasts and bone-resorbing osteoclasts. However, in pathological conditions, bone remodeling is often deregulated and the uncoupling of osteoclast and osteoblast functions may alter the extent of bone loss. Although in chronic lymphocytic leukemia (CLL), patients’ macroscopic skeletal involvement appears to be rarer than in other lymphoproliferative diseases, recent studies highlighted that the active crosstalk between leukemic B cells and bone tissue components may lead to the alteration of bone homeostasis already at early stages of the disease, becoming further evident in the advanced stages. Since the pathogenesis of bone involvement in CLL is not completely understood, this manuscript provides an overview of the clinical and biological data related to bone erosion in this disease.

**Abstract:**

Chronic lymphocytic leukemia (CLL) is the most frequent leukemia in Western countries. Although characterized by the progressive expansion and accumulation of leukemic B cells in peripheral blood, CLL cells develop in protective niches mainly located within lymph nodes and bone marrow. Multiple interactions between CLL and microenvironmental cells may favor the expansion of a B cell clone, further driving immune cells toward an immunosuppressive phenotype. Here, we summarize the current understanding of bone tissue alterations in CLL patients, further addressing and suggesting how the multiple interactions between CLL cells and osteoblasts/osteoclasts can be involved in these processes. Recent findings proposing the disruption of the endosteal niche by the expansion of a leukemic B cell clone appear to be a novel field of research to be deeply investigated and potentially relevant to provide new therapeutic approaches.

## 1. Introduction

Chronic lymphocytic leukemia (CLL) is the most frequent leukemia in Western countries, mainly occurring in the elderly and showing heterogeneous outcomes. Although characterized by the progressive expansion and accumulation of leukemic B cells in peripheral blood, CLL cells develop in protective niches mainly located within lymph nodes and bone marrow. The close interactions between malignant B cells and the surrounding tissue microenvironment play a critical role in leukemic cells’ survival and growth, as well as in drug resistance. Microenvironmental cells favoring the expansion of the B cell clone include T cells, monocytes/macrophages, nurse-like cells, endothelial cells and mesenchymal stromal cells [1,2,3]. Bone is a dynamic tissue that undergoes continuous cycles of modeling and remodeling. The balance between osteoclast and osteoblast activities ensures that bones are renewed and maintained throughout one’s lifetime. However, when the balanced activity of these cells is disrupted, bone structure results affected. It has become further evident in recent years that interactions between bone cells and immune or neoplastic cells may influence bone homeostasis, as well as change their phenotype. Indeed, bone damage is often associated with inflammatory diseases, such as rheumatoid arthritis, as well as with the migration and infiltration of neoplastic cells to the bone tissue [4,5]. We recently observed higher skeletal erosion in CLL patients than in normal controls, in particular in the progressive stages of the disease (Binet C versus A) [6]: this observation suggests that the expansion of the neoplastic B cell clone may influence and contribute to bone tissue derangement. Indeed, we demonstrated that leukemic cells impair osteoblast differentiation while stimulating osteoclastogenesis [7]. In addition, in CLL patients, the expansion of CD16+ monocytes correlates with the rate of bone erosion [8], thus suggesting that these cells are more prone to differentiate toward osteoclasts. The present review summarizes all data that evidences a potential link between the progressive accumulation of leukemic cells and bone tissue degeneration in CLL patients. Specific drugs that are currently in use for the treatment of this disease and potentially effective in counteracting these processes are also discussed.

## 2. Background on Bone Remodeling

Throughout life, both cortical and trabecular bone are gradually renewed through bone remodeling. Bone remodeling is a process in which old or damaged bone is removed by osteoclasts and replaced with new bone formed by osteoblasts. Osteoclasts are tissue-specific macrophages derived from hematopoietic stem cells (HSCs) that degrade bone via the secretion of acid and proteolytic enzymes, such as Cathepsin K [9]. Osteoblasts arise from multipotent mesenchymal precursors committed to osteoprogenitors, further differentiating into the osteoblastic lineage through the expression of the transcription factors RUNX2 and Osterix. Osteoblasts produce extracellular proteins, including osteocalcin, alkaline phosphatase and type I collagen [10]. Multiple cytokines and signaling pathways are responsible for coupling the activities of the resorbing osteoclasts to the differentiation of osteoblast precursors. The receptor activator of the nuclear factor-kB (NF-kB) (RANK)/RANK ligand (RANKL)/osteoprotegerin (OPG) axis has a key role in the bone remodeling process. RANKL, which is also called osteoclast differentiation factor (ODF) or TNF ligand superfamilymember 11 (TNFSF11), is highly expressed in osteoblasts, osteocytes, activated T lymphocytes and lymph nodes, and is considered the master cytokine required for osteoclastogenesis [11,12,13,14]. In the presence of macrophage colony-stimulating factor (M-CSF), the binding of RANKL to RANK, which is expressed on mononucleated hematopoietic precursors, stimulates their differentiation into osteoclasts, which are, by definition, multinucleated cells with at least three nuclei and are characterized by the presence of a high concentration of vacuoles, including lysosomes filled with acid phosphatase, in the cytoplasm. OPG instead acts as a soluble receptor for RANKL that competitively binds RANKL and potently inhibits osteoclastogenesis. Mice with the genetic deletion of RANK and RANKL exhibit severe osteopetrosis [15]. RANKL binding to RANK induces the trimerization of the receptor and the recruitment of adaptor proteins, such as TNFR-associated factors (TRAFs), further activating mitogen-activated protein kinases (MAPKs), NF-kB and activator protein-1 (AP-1). The subsequent signaling process is characterized by amplifying the nuclear factor of activated T-cells cytoplasmic 1 (NFATc1) via the orchestrated signaling of activated AP-1 and co-stimulatory signal-mediated intracellular Ca^2+^ oscillation. After the initial induction of NFATc1 by NF-kB, c-Fos/AP-1 and NFATc2, RANK signaling cooperates with immunoglobulin-like receptor/immunoreceptor tyrosine-based activation motif (ITAM) signals, such as TREM2/DAP12 and OSCAR/FCRγ, leading to the robust amplification of NFATc1 and its translocation to the nucleus [16]. Moreover, several pro-inflammatory cytokines, such as TNFα, IL-1β, IL-6, IL-8 and IL-17, are known to favor osteoclastogenesis, while others, such as IL-3, IL-4, IL-10, IL-13 and IFN, are considered anti-osteoclastogenic [17,18]. Also, several chemokines may influence bone resorption and bone formation through autocrine and paracrine mechanisms. Among these CCL3, CXCL8 and CCL17 are pro-osteoclastogenesis, and CCL20 not only stimulates osteoclast differentiation but also osteoblast differentiation. Several studies showed that the CXCL12/CXCR4 axis is involved in both osteoclast differentiation and osteoblast formation [19]. Bone homeostasis is therefore tightly regulated by local and systemic factors, including the body’s innate and adaptive immune cells, via direct interaction and an intermediate cellular response. In pathological processes, the unbalanced production of these factors or altered cell-to-cell interactions may thus lead to bone derangement.

## 3. Bone Tissue Erosion in Chronic Lymphocytic Leukemia Patients

Although macroscopic skeletal involvement was previously considered rare in CLL, several cases of patients presenting osteolytic lesions were more recently reported. Indeed, after a full literature revision, Bacchiari and co-workers concluded that bone lesions appear to be not-so-rare events [20]. In this study, the authors observed that in 11 out of 22 cases described in the literature, the osteolytic lesions were localized in the axial skeleton or proximal long bones, while only in rare cases, they were localized in the skull or facial bones. Moreover, multiple fractures were observed in eight cases. It is of interest to note here that the authors stated that in 13 CLL cases described, the patients developed bone metastasis/symptomatic bone lesions as the first presentation of the disease [20]. In addition, hypercalcemia appeared to be frequently associated with osteolysis and mostly related to Richter’s transformation or co-occurring with multiple myeloma [20]. However the pathogenesis of bone involvement in CLL is still not completely understood, and deeper investigations could be of help to clarify the underlying mechanisms causing bone remodeling under the influence of the expansion of malignant B cells. In a previous retrospective study, F. Fiz and collaborators observed structural skeletal alterations in advanced CLL patients: a significant trabecular bone volume enlargement, paralleled by a decrease in the compact bone volume, was more evident in patients with respect to controls, as quantified using the computational analysis of CT images [21]. Structural bone alterations were particularly evident in the appendicular bones, and, interestingly, the degree of bone erosion appeared significantly related to a poor outcome [21]. This finding may suggest that radiologic risk assessment could be useful to predict disease aggressiveness and to better tailor patient-specific treatment protocols. One more study was therefore performed to specifically clarify whether bone erosion characterized only patients in the advanced stage or also those at an earlier disease stage [6]. A cohort of 36 treatment-naive CLL patients (16 Binet A, 12 Binet B, 8 Binet C) were enrolled to analyze their skeletal structure and bone marrow distribution using a computational approach to PET/CT scan images. Skeletal alterations were observed in all risk classes, apparently independent from Binet stages, when the whole skeleton was analyzed. However, a correlation with the clinical disease stage emerged when the appendicular districts were examined: shaft cortical thinning progressively increased with a raise in the clinical index of disease severity from Binet A to Binet C and appeared related to the number of RANKL+ CLL cells. PET-FDG imaging found that the same long bone shafts were colonized by metabolically reactive bone marrow (RBM), thus suggesting that CLL cells may contribute to skeletal derangement, promoting osteoclast differentiation. The experiments performed in a xenograft NOD-SCID-γ-null mouse model further confirmed that the administration of CLL cells was associated with the thinning of the femoral cortex. Moreover, this in vivo model supported the hypothesis that the activation of the RANK/RANKL is involved in bone erosion by CLL cells. The anti-RANKL mo-Ab Denosumab was in fact capable of sparing leukemic cells since the number of neoplastic B-cells detected in the bone marrow (BM) and spleen was significantly higher in untreated than in treated mice [6].

## 4. CLL Cells Affect Osteoblast and Osteoclast Differentiation

**Figure 1:**
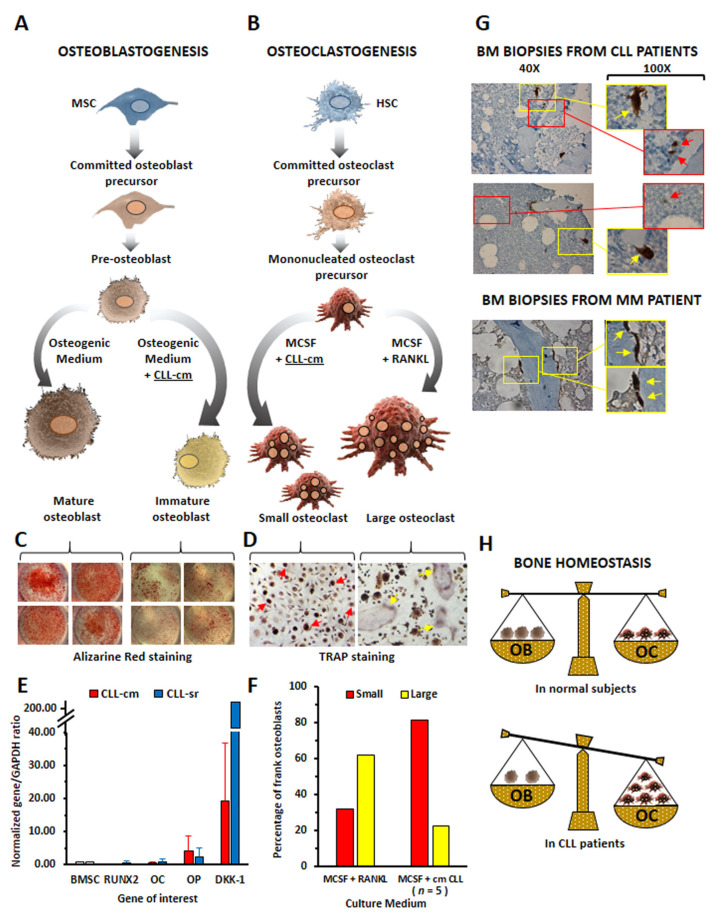
Comparison of osteoblastogenesis and osteoclastogenesis in normal and CLL bone tissue. (**A**,**B**) schematic representation of in vitro induced MSC osteoblastogenesis or HSC osteoclastogenesis, with/without the addition of CLL-conditioned medium (CLL-cm). (**C**) The addition of CLL-cm to osteoblast precursors inhibited deposition of mineralized matrix (Alizarine red staining). (**D**) RANKL-dependent pre-activation was necessary to induce the formation of mature large osteoclasts (Alkaline phosphatase tartrate-resistant activity detection (TRAP)). Red arrows indicate small osteoclast-progenitors, while yellow arrows indicate large multinucleated mature osteoclasts. Magnification are 40X and 100X, as indicated. (**E**) Modulation of the levels of expression of RUNX2, Osteocalcin (OC), Osteopontin (OP) and DKK-1 in osteo-induced BMSC cultured with CLL-cm (red bars) or CLL-sera (blue bars). Data represent the mean of 10 CLL patients evaluated using quantitative RT-PCR and normalized to the housekeeping gene (GAPDH) and to BMSC used as control. (**F**) The addition of CLL-cm to osteoclast-induced monocytes enhanced the number of osteoclast precursors (small trinucleated TRAP+ cells) but RANKL appeared necessary to allow for a complete osteoclast maturation (large multinucleated TRAP+ cells). (**G**) In bone biopsies from CLL patients (*n* = 2), the presence of small (red enlargements and arrows) and large (yellow enlargement and arrows) TRAP+ osteoclasts was evident instead of being homogeneously large in multiple myeloma (MM) biopsy. (**H**) Resulting imbalance of bone homeostasis in CLL patients versus normal subjects. (Graphics and images of live cells displayed here were not previously published.).

Ex vivo co-cultures of CLL cells with differentiating osteoblasts/osteoclasts helped us to clarify the potential role played by leukemic cells in bone tissue derangement in the disease [7]. BMSCs from healthy donors and differentiated toward osteoblasts in an osteogenic medium did not reach complete maturation upon co-culture with CLL cells or with the addition of CLL-cell-derived conditioned media (CLL-cm). The inhibition of osteoblast differentiation was documented by decreased levels of RUNX2 and osteocalcin mRNAs, increased osteopontin and DKK-1 mRNA levels, and a marked reduction in mineralized matrix deposition (Figure 1A,C,E). CLL-cm added to the medium culture instead enhanced the differentiation of normal monocytes toward osteoclasts. However, the presence of exogenous RANKL in the induction phase of differentiation appeared necessary to generate a high number of large multinucleated cells that were fully competent in resorbing the bone surface (Figure 1B,D,F). When CLL-cm was added without previous RANKL activation, healthy monocytes could only differentiate toward a state of osteoclast precursors (small trinucleated cells) [7]. Indeed, previous observations from Chappard D. and Rossi J.F. and co-workers are consistent with our in vitro data [22,23]. Histologic and electron microscopy studies from these authors showed that B cell malignancies presenting bone alterations, including CLL, displayed a discrete number of osteoclasts, identified as tartrate-resistant acid phosphatase (TRAP)+ cells and close to bone trabeculae, but smaller than those detected in multiple myeloma (MM): these cells induced areas of micro-resorption and appeared very close to malignant lymphoid cells. The cytomorphometric analysis of bone biopsies further confirmed the heterogeneity in the size of TRAP+ cells, showing a bimodal distribution [24].

The presence of a heterogeneous population of TRAP+ cells within the BM of CLL patients was also evident in bone biopsy sections derived from CLL cases and examined in our study [7] (Figure 1G). Collectively, the in vivo and in vitro observations may indicate that altered bone homeostasis can be a feature of CLL (Figure 1H) and that a higher number of active small osteoclasts can be necessary to cause a rate of resorption similar to those observed in MM patients. It is further known that MM malignant B cells may release high amounts of RANKL, while in CLL, the shedding of RANKL by leukemic cells was rarely found [25]. This observation may suggest that, initially, CLL-monocytes may differentiate toward osteoclastic precursors under the influence of cytokines secreted by leukemic or microenvironmental cells, but then fail to reach complete maturation due to low RANKL levels. The study of Borge M. and collaborators [26] provides support to this hypothesis. Here, the authors described the case of a 72-year-old CLL patient showing extensive lytic bone lesions at X-ray and MRI examinations, a diffuse infiltrate of small mononuclear cells with features of CLL cells (CD20+, CD23+, CD5+, CD138−) in a bone biopsy, and the presence of high levels of RANKL in plasma (888 pg/mL). In order to clarify whether high concentrations of soluble RANKL might contribute to bone damage, the authors demonstrated that the addition of 10% of the plasma from this patient to the in vitro culture of the monocytic cell line THP1 significantly stimulated osteoclast formation. At difference with other CLL cases tested, the amount of RANKL released by purified CD19+ leukemic B cells was already detectable in basal conditions (102 pg/mL) and aberrantly increased after CPG activation (1600 pg/mL). Therefore, RANK/RANKL interaction, as well as the overproduction of some cytokines by CLL cells, such as IL-8, TNFα and IL-6, may influence bone metabolism, further creating interactive niches sustaining leukemic cell proliferation. The importance of the RANK–RANKL interaction as a microenvironmental signal promoting CLL cell development and survival in the murine and human systems was also recently highlighted by expressing a human lymphoma-derived RANK^K240E^ variant in mice B lymphocytes [27]. B cell-intrinsic RANK^K240E^ drove a fully penetrant systemic lupus erythematosus (SLE)-like disease and facilitated B cell transformation and CLL development, which was not driven by an altered expression of the mutated RANK but rather its aberrant signaling in response to microenvironmental RANKL. Moreover, murine B cells expressing the RANK variant survived in vitro significantly better than their wild type counterparts cells in in vitro settings, even in the absence of exogenous stimulation. RANKL stimulation, on the other hand, induced more pronounced JNK, ERK and PI3K/AKT activation in the transgenic cells, resulting in higher expressions of the antiapoptotic molecule Bcl-2 and cell cycle regulator Cyclin-D1 [27].

## 5. Cytokines Produced by Neoplastic B Cells Are Involved in Bone Tissue Derangement of CLL Patients

Beyond certain co-stimulatory molecules, such as immunoreceptor tyrosine-based activation motif (ITAM) adaptors DAP12 and FcRγ that contribute to amplifying osteoclast differentiation [28], some proinflammatory cytokines may also stimulate osteoclast activation or impair osteoblast differentiation. The unbalanced production of certain cytokines appears strictly associated with bone disease pathogenesis. TNFα plays an important role in chronic inflammatory diseases, such as rheumatoid arthritis (RA) [29]. Here, bone marrow mesenchymal cells (BMSCs) appear primarily involved in joint damage [30], and the increased local production of TNFα may injure the BM microenvironment, thus affecting the reserves of hematopoietic progenitor cells [30,31]. Other cytokines, such as those belonging to the interleukin-6 (IL-6) family (i.e., IL-6, interleukin-11 and oncostatin M), or interleukin-1 and -8, may regulate bone structure and function; it is therefore known that their over-expression can be involved in the pathogenesis of certain bone diseases or bone metastatic cancers [32,33,34]. In our experimental model of CLL-conditioned osteoblast and osteoclast differentiation, we demonstrated reduced osteoblastogenesis and increased osteoclastogenesis by TNFα, IL-6 and IL-11, which were released in the medium culture by leukemic B cells [7]. While TNFα appeared to be involved in driving the osteoclasts toward a complete maturation stage, IL-6 and IL-11 were instead related to the generation of osteoclast progenitors. It is worth noting that malignant CLL cells release TNFα [35], and the levels of plasma TNFα, which were higher in CLL patients than in healthy controls, correlated with disease stage, CD38 expression and chromosomal abnormalities (17p and 11q deletion) [36]. Interestingly, TNFα levels were significantly higher in the sera from patients with advanced disease than in patients with stable disease and appeared directly proportional to the degree of appendicular bone erosion [6]. The imbalanced production of particular cytokines, together with the enhanced expression of RANK and RANKL by leukemic B cells, may therefore affect bone homeostasis, leading to increased bone resorption, further causing bone damage in progressive disease CLL cases.

## 6. Chemokines: Can They Be Additional Players in CLL Bone Remodeling?

Chemokines and their receptors are pivotal in CLL trafficking, homing and interactions between neoplastic and microenvironmental cells. Leukemic CLL cells, as well as CLL-associated stromal cells, release various chemokines involved in shaping their microenvironment [37,38,39,40]. Bone resorption is elicited by CCL3, CCL4, CCL17 and CXCL8, which are also known to be released by CLL cells, constitutively or after activation. The CXCL12/CXCR4 interaction, which is relevant in inducing CLL cell survival, proliferation and chemotaxis, also has remarkable influences on bone remodeling: through CXCR4, CXCL12 binding promotes osteoclastogenesis and induces osteogenic differentiation of mesenchymal stromal cells in cooperation with bone morphogenetic protein (BMP) signaling [37,41,42]. CXCL9, which is found at high concentrations in the sera of CLL patients and supposedly produced by stromal and activated B cells, also interferes with bone formation, further possibly favoring osteoclast differentiation and resorption [19,43,44,45,46,47]. Given the evidence of the interferences of these chemokines in bone homeostasis [17] and their simultaneous release by CLL or stromal cells, it is feasible to foresee their possible participation in patient bone remodeling. From this perspective, the inhibitors of chemokines or their receptors, alone or in combinatorial protocols, might contribute to directly and/or indirectly counteracting B cell clone expansion, as well as their crosstalk with microenvironmental cells, as demonstrated with the anti-CXCR4 mo-Ab ulocuplumab in CLL/stromal cells co-cultured in vitro [48]. Although, to date, no literature data directly support this hypothesis, novel studies in this field could improve our knowledge of the crosstalk mechanisms among these different cell types within the endosteal niche in the disease.

## 7. Monocyte Polarization by Leukemic B Cells and Bone Tissue Remodeling in CLL Patients

**Figure 2:**
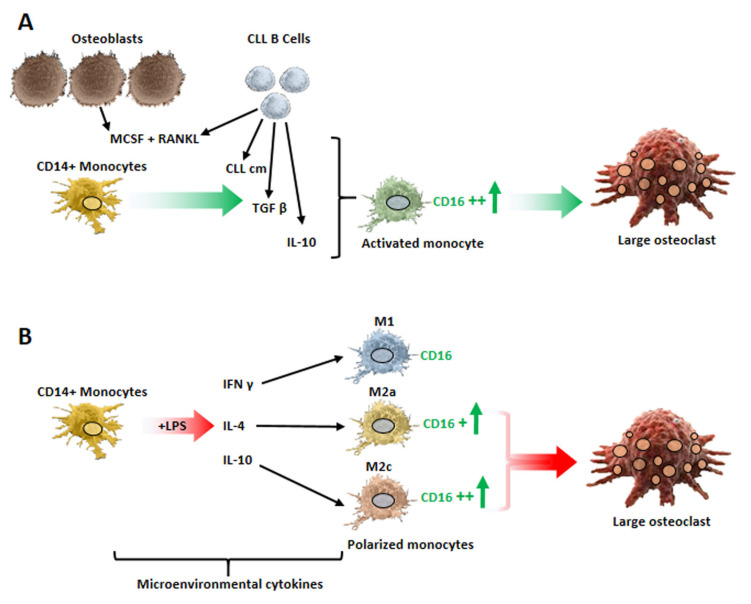
Cytokines produced by leukemic B cells or present in the microenvironment may drive monocyte differentiation, leading to enhanced osteoclastogenesis. (**A**) CLL-cm (containing TGFβ and IL-10), as well as exogenously added TGFβ and IL-10, upregulates the expression of the RANK co-receptor CD16 in monocytes, thus eliciting osteoclastogenesis. (**B**) IL-4 and IL-10 activation, which polarizes monocytes toward M2a and M2c subtypes and simultaneously enhances CD16 expression, favors complete osteoclastogenesis [8]. (Graphics displayed here were not previously published).

Monocytes/macrophages assume a critical role in the maintenance and progression of CLL cells. Many studies highlighted that neoplastic B cells shape the phenotypical and functional features of the monocytes/macrophages of the CLL microenvironment. The crosstalk between leukemic B cells and monocytes leads to the polarization of monocytes toward an immunosuppressive M2 phenotype, simultaneously enhancing the survival and expansion of CLL cells. Moreover, a particular population of myeloid cells, namely, nurse-like cells (NLCs), was described in this disease. NLCs are cells of monocytic origin that spontaneously differentiate in vitro in high-density cultures of CLL peripheral blood mononuclear cells (PBMCs) [49,50]. These cells support leukemic B cell survival, further creating a permissive microenvironment [51,52]. Importantly, NLCs were also observed in vivo in the lymphoid organs of patients with CLL [50]. Since their original description [49], NLCs/CLL cell co-cultures have been used extensively to dissect key cellular and molecular interactions between leukemic B cells and their microenvironment. We reported that NLCs and CLL monocytes display features of the alternative type-2 subset (M2) [53]. NLCs are characterized by high CD11b, CD163, CD206, HLA- DR and c-MET expression and by the dysregulation of genes involved in immunocompetence [53,54,55,56]. In addition, we demonstrated that NLCs and CLL monocytes showed higher expressions of indoleamin 2,3 dioxygenase (IDO) than monocytes from normal controls [53], and IDO is regarded as a key endogenous immunologic checkpoint with a pivotal impact on tumor-associated immune tolerance [57]. In line with their immunosuppressive phenotype, CLL monocytes or NLCs significantly inhibited T cell proliferation, and this inhibition was counteracted by the concomitant addition of neutralizing anti-TGFβ or -IL-10 antibodies or IDO inhibitors in cultures. Indeed, healthy monocytes upregulated IDO after their co-culture with CLL cells [53]. Interestingly, IDO also appeared upregulated in NLCs cultured in hypoxic conditions [58]. Jitschin R. and collaborators [59] further reported that untreated CLL patients show a significantly increased frequency of monocytes CD14 + DRlow, which are defined as myeloid-derived suppressor cells (MDSCs) and characterized by high IDO levels expression, that induced the suppression of T cell activation while expanding T regulatory cells (Tregs); the MDSC-mediated modulation of T cells was attributed to their increased IDO activity. The presence in CLL patients of a higher percentage of MDSCs characterized by immunosuppressive features and expressing IDO together with others immunoregulatory molecules/cytokines, such as arginase 1 (ARG1), nitric oxide synthase (NOS2), TGF-β and IL-10, was also described by Zarobkiewicz M and co-author [60]. It is of further interest to note that the kynurenine–tryptophan ratio, which reflects increased IDO activity, was found to be higher in sera from CLL patients than in normal donors [61]. Maffei and co-authors [62] also observed the functional and phenotypic deregulation of monocytes in CLL patients: the gene expression profile analysis of CLL monocytes compared with monocytes from healthy donors, other than suggesting the deregulation of genes involved in phagocytosis and inflammation, evidenced the ability of CLL B cells to “educate” these cells, skewing them toward an immunosuppressive phenotype. Using cytofluorimetric analysis, these authors further evaluated the proportions of the three subtypes of monocytes present in CLL PBMCs: classical (CD14+ CD16−), intermediate (CD14+ CD16+) and non-classical (CD14+/− CD16++). In contrast with normal donors, they observed a significant increase in the intermediate and non-classical populations and a reduced percentage of classical monocytes. Of further note is the finding that healthy monocytes, co-cultured with CLL cells or with their conditioned medium, were induced to upregulate CD16, which is the Fcγ type III low-affinity receptor for IgG (FcγRIIIa). A higher number of monocytes expressing CD16 in CLL patients was also described by Kowalska and co-authors [63]. Bolzoni M and co-workers [64] further interestingly reported that sorted bone marrow CD14+ CD16+ cells from myeloma patients were more pro-osteoclastogenic than CD14+ CD16− cells in cultures ex vivo. Additionally, it was demonstrated that the number of bone marrow CD14+ CD16+ cells was higher in patients with active myeloma than in those with monoclonal gammopathy of undetermined significance [64]. CD16 is a RANK co-receptor, contributing to the amplification of osteoclast differentiation [65]; this evidence, along with the previous observations, prompted us to evaluate, first, the percentage of monocyte subtypes in a cohort of PBMCs from 35 CLL patients, and second, whether the eventual expansion of a particular subset could be related to the enhanced rate of bone erosion previously reported [8]. We confirmed that the percentages of the two subsets expressing CD16 (intermediate and non-classical) were significantly higher in CLL cases than in PBMCs from normal donors, and we found a direct correlation between the percentage of intermediate monocytes and the levels of bone erosion. We also demonstrated that the percentage of healthy monocytes expressing CD16, and to a lesser extent RANK and RANKL, was significantly enhanced when they were cultured with CLL-cm alone, IL-10 or TGFβ. It is worth noting that a higher number of monocytes expressing CD16 appeared capable of generating a higher number of large osteoclasts, and, indeed, the addition of an anti-CD16 neutralizing antibody counteracted osteoclast differentiation. We further demonstrated that monocytes polarized toward the M2 phenotype, in particular M2c, were more prone to differentiate toward osteoclasts [8]. In agreement with our observations, Jihyun Yang et al. [66] observed that M2 monocytes differentiated into osteoclasts more efficiently than M1, especially when pre-activated with IL-10. Gene expression profile analysis of public data showed that the key osteoclastogenic transcription factor NFATC1 was also significantly higher in M2 versus M1 monocytes in CD16+ versus CD16- monocytes and in intermediate and non-classical versus classical monocytes, further supporting the results from our experiments [8]. Collectively, these findings suggest that the expression of CD16 facilitates the differentiation of monocytes toward osteoclasts and that CD16 could represent a marker of osteoclast precursors in CLL, as was previously indicated for psoriatic arthritis [67]. It is also worth noting that, in particular contexts, myeloid-derived suppressor cells (MDSCs) may differentiate toward osteoclast precursors, as demonstrated in breast cancer, rheumatoid arthritis or multiple myeloma [68,69,70,71,72,73]. The observations presented here are schematically summarized in Figure 2.

The above-reported data may suggest that myeloid cells, expanded under the influence of leukemic B cells and acquiring an immunosuppressive phenotype, may directly or indirectly contribute to bone derangement in CLL patients: together with the production of cytokines, such as TGFβ and IL-10, the upregulation of the FcγRIIIa (CD16) antigen also appeared to contribute to the amplification of osteoclasts differentiation.

## 8. Disruption of the Endosteal Niche in CLL Patients: Cause and Effect of the CLL Clonal Expansion?

Hematopoiesis and bone remodeling take place in the same niche, namely, the endosteal one. Various cell types, such as adipocytes, fibroblasts, osteoclasts, osteoblasts and vascular cells, contribute to these processes by communicating with each other [74]. However, this niche is also the site of leukemic B cell infiltration, either during disease progression or relapse where the neoplastic and bone cells come into close contact.

We demonstrated that partially or fully induced osteoclasts supported the viability of CLL B cells and stimulated the proliferation of a limited number of leukemic cells (Ki67+) [7]. In turn, CLL cells inhibit osteoblastogenesis and stimulate osteoclastogenesis through the release of particular cytokines or possibly the expansion of myeloid subsets (M2, MDSC), finally creating support for their survival through the enhancement of osteoclast activation. Taken together, these results suggest that the disruption of the endosteal niche appears to be at the same time cause and effect of the expansion of the leukemic B cell clone. It is well known from literature data that neoplastic CLL cells shape their microenvironment by subverting the primary function of the immune cells [75]: our findings demonstrate that similar effects are also exerted by leukemic cells on the major cell types of the bone tissue.

Here, we would like to further highlight that adipocytes also could take part in bone derangement, since the number of bone marrow adipocytes (BMAs) increases with age and CLL is a disease of the elderly. Increased BM adiposity may inevitably result in impaired osteoblastic bone formation due to competitive lineage allocation between the adipogenesis and osteogenesis of BMSCs. Indeed, bone marrow adipocytes specifically secrete chordin-like1 (Chrdl1) and gremlin1 (Grem1), which significantly suppress bone formation [76]. Moreover, several transcription factors, such as peroxisome proliferator-activated receptor γ (PPARγ) or CCAAT/Enhancer binding protein α and β (C/EBP α and β), which are required for BMSC adipogenesis, are expressed in HSC and promote osteoclastogenesis [76]. The adipokines secreted by BMA, such as TNFα and adiponectin, also regulate both osteoblast and osteoclast differentiation [77]. In addition, BMAs produce a low quantity of RANKL and MCSF that may contribute to osteoclastogenesis. In obese individuals, adipocytes regulate bone integrity via the secretion of adipokines [78] and may induce osteoporosis [79]. Liu and collaborators [80] interestingly observed that malignant myeloma cells reprogram BMAs, leading to the enhancement of osteoclastogenesis and the suppression of osteoblastogenesis through methylation and the downregulation of PPARγ. To date, detailed molecular mechanisms accounting for enhanced BM adipogenesis during pathologic bone loss still need to be explored, but in the near future, it can be expected that this research field will provide grounds for novel biological and therapeutic implications.

## 9. Impact of BCR Signaling Inhibitors on the CLL Endosteal Niche

Therapeutic regimens in CLL have undergone tremendous changes in recent years. The BCR signaling pathway is involved in the pathogenesis of CLL, and drugs that inhibit Bruton’s tyrosine kinase (BTK) and phosphoinositide 3′-kinase (PI3K) are the standard of care when treating CLL. The inhibitor of BTK, namely, Ibrutinib, blocks B-cell receptor signaling, driving CLL cells into apoptosis and disrupting cell migration or adherence to the protective microenvironment. Moreover, the evidence that BTK is also expressed on many hematopoietic cells suggests that the huge success of the BTK inhibitors in CLL also appears to be related to its pleiotropic activity on cells of the tumor microenvironment [81,82,83]. It has been, for example, recognized that Ibrutinib reshapes the T-cell compartment in CLL toward improved antitumor function, potentially shifting Th2 vs. Th1 polarization in CLL patients [84,85]. Ibrutinib further reduces the expression of inhibitory receptors, such as PD1 [86]. In addition, ibrutinib not only inhibits the secretion of chemokines, such as CCL3, CCL4, CXCL12 and CXCL13, from CLL cells and their supporting cells but also inhibits the TLR signaling pathway.

Bruton Tyrosine Kinase (BTK) integrates RANK/RANKL and ITAM pathways along osteoclast formation and activity [65]. Shinohara et al. [87] reported that Ibrutinib downregulates the expression of NFATc1, which is the key transcription factor for osteoclastogenesis and disrupts the formation of the actin ring in mature osteoclasts. These authors further demonstrated that the oral administration of ibrutinib protects against bone loss in a mouse model of osteoporosis, suggesting that this BTK inhibitor is a potential therapeutic agent for certain osteoclast-related diseases. Evgenii Shumilov et al. [88] described the case of a 44-year-old male patient who developed spontaneous bilateral humerus fractures due to osteolytic lesion 3 years from diagnosis of the CLL following refractoriness to previous immunochemotherapy: fourth-line therapy with Ibrutinib was interestingly able to achieve long-lasting remission, further preventing the manifestation of new osteolytic lesions. We observed that Ibrutinib was capable of significantly counteracting the in vitro generation of large mature osteoclasts, which was enhanced by the addition of CLL-cm in the culture of RANKL-activated monocytes [7]. The activity of Ibrutinib on monocytes/macrophages is, however, not yet completely clear. On the one hand, it was shown that Ibrutinib disaggregates macrophage/CLL cell interactions in BM [89], while on the other hand, in vitro studies demonstrated that Ibrutinib enhances M2 properties in NLCs, thus leading to the inhibition of phagocytosis, upregulation of CD163 and CD206, and modulation of a peculiar cluster of genes regulating immune suppression. Furthermore, Ibrutinib, by reinforcing IL-10 production by NLCs, did not totally halt IL-10-mediated survival signals on CLL cells [90]. In line with these data, Boissard and collaborators previously suggested that NLCs contribute to mediating resistance to Ibrutinib in CLL patients [91]. NLCs’ development appears to be dependent on MEK/ERK signaling [92] and it is possible to suppose that the efficacy of Ibrutinib on monocytes may depend on their differentiation stage, as well as on different routes engaged along signaling pathways that lead to different lineage commitment. It is worth noting that the evaluation of long-term immunophenotypic and quantitative changes in circulating immune cells in patients with CLL, over 4 years of first-line treatment with Ibrutinib, demonstrated that over time, Ibrutinib results in improvements in innate and adaptive immunity; in particular, there was a decline in immunosuppressive cells, such as Tregs and MDSCs, and a restoration of classical monocytes, typically phagocytic, versus non-classical monocytes [93].

Among the second generation of BTK inhibitors that were more recently deeply explored for CLL treatment, Acalabrutinib and the dual BTK/TEC inhibitor Tirabrutinib also appear capable of inhibiting osteoclasts generation [94,95]. Yeon JT and co-workers [96] further reported that Idelalisib, which is the inhibitor of PI3Kδ, inhibited RANKL-induced osteoclast differentiation: stimulation with RANKL upregulated PI3Kδ expression on bone-marrow-derived macrophages, and Idelalisib could therefore block the PI3Kδ-Akt-c-Fos/NFATc1 signaling cascade, thus counteracting differentiation and pre-osteoclast migration. These findings appear to corroborate the suggestion that these inhibitors not only counteract the growth of leukemic B cell clones directly by blocking BCR activation and signaling but also indirectly by acting on microenvironmental cells, such as those composing the focused bone, which favors their survival.

## 10. Conclusions and Future Directions

Collectively, the above-discussed observations demonstrate that bone tissue actively interacts with the CLL clones throughout all the disease stages. Support for the survival and expansion of leukemic B cells may take place and exert the most relevant biological effects in the initial phases of the pathology. Once the crosstalks between resident and leukemic cells in the microenvironmental niche have been established, more consistent alterations of the bone homeostatic mechanisms can be observed. This hypothesis could explain why the surmised clinical bone alterations are more evident in late-stage patients. In this respect, further studies corroborated by CT and/or MR imaging of CLL patients could be of help in predicting the stage and course of the disease. Moreover, an apparent good novelty is the body of data that suggests that the BTK and PI3K inhibitors, which are currently in use to treat the disease, are also effective on the tumor microenvironment, additionally limiting the disease-associated bone lesions.
